# Primary hyperparathyroidism during pregnancy: ultrasound as an accurate preoperative localization imaging modality

**DOI:** 10.1186/s13023-024-03519-w

**Published:** 2025-03-31

**Authors:** Mengyuan Zhou, Yudi He, Yanwen Luo, Ou Wang, Quan Liao, Yuxin Jiang, He Liu, Qingli Zhu

**Affiliations:** 1 Department of Ultrasound, Peking Union Medical College Hospital, Chinese Academy of Medical Sciences and Peking Union Medical College, Beijing, 100730 China; 2 Key Laboratory of Endocrinology, Department of Endocrinology, Peking Union Medical College Hospital, National Commission of Health, Chinese Academy of Medical Science, Beijing, China; 3 Department of General Surgery, Peking Union Medical College Hospital, Chinese Academy of Medical Sciences and Peking Union Medical College, Beijing, China

**Keywords:** Primary hyperparathyroidism, Pregnancy, Parathyroid, Ultrasound, Radionuclide

## Abstract

**Background:**

Accurate identification of parathyroid lesions in primary hyperparathyroidism (PHPT) patients is essential for minimally invasive surgery during pregnancy.

**Materials and methods:**

Patients who were diagnosed with PHPT during pregnancy and who had undergone surgical treatment between January 2005 and September 2023 were retrospectively included. Demographic and clinical characteristics and preoperative parathyroid ultrasound (US) and technetium-99m sestamibi (^99m^Tc-MIBI) scintigraphy results were collected. Histopathologic examinations were conducted for all lesions removed during neck surgery, and the results were considered as the reference standard.

**Results:**

A total of 19 pregnant patients with PHPT who had undergone parathyroidectomy were retrospectively included in the study. The median age was 30 years. Sixteen (16/19, 84.2%) patients had single-gland disease and three (15.8%) had two lesions. Three patients were confirmed as multiple endocrine neoplasia type 1. The median size of all lesions was 1.8 cm (0.6–7.5 cm). All patients had undergone US examination, and eight patients had ^99m^Tc-MIBI scintigraphy. A total of 21 lesions were found on US. The diagnostic sensitivity of the US was 95.45% per lesion and 100% per patient. One lesion, with a maximum diameter of 0.6 cm, was missed preoperatively by either US or ^99m^Tc-MIBI scintigraphy. Nine patients had surgery in the second trimester and 88.89% of them had a full-term pregnancy after surgery. There were no complications in the newborns.

**Conclusions:**

In pregnant PHPT patients, US achieved high sensitivity for preoperative lesion localization. Surgery during the second trimester after accurately localizing the lesion(s) by US improved the patients’ pregnancy outcomes.

**Supplementary Information:**

The online version contains supplementary material available at 10.1186/s13023-024-03519-w.

## Introduction

Primary hyperparathyroidism (PHPT) in pregnancy is rare, accounting for roughly 1% of the total PHPT patients. However, PHPT in pregnancy can increase the risk of maternal and infant complications, including hyperemesis gravidarum, pre-eclampsia, neonatal hypocalcemia convulsion, even life-threatening complications such as abortion or stillbirth, and neonatal death [1–4]. It is important to early recognize the entity because parathyroidectomy during mid-pregnancy has been proven to improve the prognosis of pregnant women with PHPT [5–7]. The precise preoperative location of the lesions is crucial for the success of the surgery.

Widely accepted first-line preoperative localization imaging examinations include neck ultrasound (US) and technetium-99m sestamibi (^99m^Tc-MIBI) scintigraphy [8, 9]. However, the choice of imaging protocol was essentially based on the following elements in a pregnant woman: reduction of radiation of the fetal thyroid and whole body as low as reasonably possible, and ensuring the most sensitive imaging possible [10, 11]. US has been widely used for preoperative evaluation because of the advantages of non-radiation and ease of use and convenience. However, there is still a lack of evidence on whether US can be accurately used as the single imaging modality for preoperative localization with the intent to minimize nuclide exposure in pregnant patients.

Our study aimed to evaluate the diagnostic performance of parathyroid US as the primary imaging modality in evaluating PHPT in pregnant women. Our secondary aim was to evaluate the added benefit of parathyroid radionuclide imaging.

## Materials and methods

### Study design and patients

In this study, we retrospectively included consecutive patients from our institution diagnosed from January 2005 to September 2023 with PHPT during pregnancy. The inclusion criteria were PHPT patients with pregnancy who had undergone surgery and had the definite pathological results. If patients did not have preoperative US examination, and/or 6-month postoperative laboratory values, they were excluded from analysis.

PHPT was defined as hypercalcemia with parathyroid hormone (PTH) levels higher than normal or not suppressed; or normocalcemic PHPT as a persistently normal total albumin corrected and ionized serum calcium concentrations and persistently elevated PTH levels after ruling out secondary causes of hyperparathyroidism such as renal disease, hypovitaminosis D, and hypercalciuria [12]. 

The diagnosis of PHPT with pregnancy was determined as PHPT diagnosed during pregnancy or pregnancy occurring in PHPT patients with uncontrolled conditions [10]. 

### Clinical information

The clinical charts for each patient were reviewed to collect the following information: age, clinical symptoms, biochemical data, pathology results, and pregnancy outcomes. In addition, the postoperative laboratory values were collected including PTH and blood calcium levels at least 6 months after parathyroidectomy. The pregnancy outcomes were also obtained.

### Imaging protocol and interpretation

Preoperative parathyroid US evaluations were performed by radiologists with at least five years of experience in parathyroid US. Evaluations were performed using one of three US machines (IU22 or Epiq, Philips, Amsterdam, North Holland, Netherlands; LOGIQ E9, GE, Boston, Massachusetts, USA) and a broadband linear array transducer (L12–5 MHz, Philips; ML6-15, GE). For each suspicious lesion identified, longitudinal and transverse views were obtained using grayscale and Color Doppler modes. The suspicious lesions were described based on location (left upper, left lower, right upper, right lower, and ectopic location), size (length, width, and thickness in centimeters), shape (regular/irregular), boundary (clear/ uncircumcised), echo pattern (hyperechoic/isoechoic/hypoechoic compared to the thyroid gland), and blood flow (abundant: 3 or more blood signals in the same section; medium: 1–2 blood signals; none).

The planar images and SPECT/CT data were reviewed by an experienced nuclear medicine doctor to determine the existence of abnormal radioactive uptake and location of the suspected lesions. The injection dose of ^99m^Tc-MIBI scintigraphy (Atom Hitech Co., Ltd.) was 740 ± 20 MBq for each patient. The planar images were obtained 20 min and 2 h after the intravenous administration using a γ-camera with a pinhole collimator (200,000 counts during the two-phase acquisitions). ^99m^Tc-MIBI scintigraphy was performed on a 64-slice Philips Precedence device (SPECT: low-energy high-resolution collimator, 128 × 128 matrix, zoom factor of 1.0; CT: 120 kV, 30 mAs, slice thickness of 3 mm). The planar images and SPECT/CT data were reviewed by an experienced nuclear medicine doctor to determine the existence of abnormal radioactive uptake and location of the suspected lesions.

Although studies have shown that ^99m^Tc-MIBI scintigraphy is safe for pregnant patients with PHPT, and lesions can be found even with half the dose, the potential radiation exposure to the fetus must be considered [13]. Per the policy at our institution for PHPT, parathyroid US was recommended as the first-line imaging examination.

After a thorough communication, the patient would decide whether to undergo ^99m^Tc-MIBI scintigraphy.

### Surgery and histopathological examinations

Surgeries were performed after completing the necessary examinations and were based on a multidisciplinary decision. If lesions were discovered during early pregnancy or the clinical situation became unstable, the patient underwent surgery after the abortion or labor was induced. If the clinical situation was stable, the patients underwent surgery during the second trimester or after delivery. The location and size of the parathyroid lesions were recorded during the surgeries. Histopathological examinations were conducted for all lesions removed during the neck surgery and the histopathological results were considered as the gold standard.

### Statistical analysis

We used SPSS version 25.0 software (IBM Corporation, Armonk, NY, USA) for statistical analysis. Significance was set at two-sides *p* < 0.05. The measurement data (age, free blood calcium, blood phosphorus, blood potassium, parathyroid hormone) were described by M (Q25, Q75). The pathological type (adenoma, hyperplasia, adenocarcinoma, and atypical adenoma), reason for seeking medical advice (bone involvement symptoms, urinary system symptoms, gastrointestinal symptoms, others), and ultrasonic manifestations (internal echo, shape, boundary, blood flow, etc.) were compared with each other using frequency tables.

## Results

### Clinical information and pregnancy outcomes

A total of 20 patients with PHPT during pregnancy were initially involved. One patient was excluded due to a lack of detailed surgical and pathological results (*N* = 1). Ultimately, 19 patients clinically diagnosed with hyperparathyroidism during pregnancy were included for further study (Tables 1 and 2).

**Table 1:** Patient, lesion, and imaging characteristics in 19 pregnant patients with PHPT

Parameter	Number
Age	
Mean	31.58 ± 4.27
Median	30 [26–44]
≤ 30	10/19 (52.63)
>30	9/19 (47.37)
Classification	
MEN1	3/19 (15.79)
Multiple parathyroid lesions	3/19 (15.79)
Clinical manifestations	
Gastrointestinal symptoms	12/19 (63.16)
Bone involvement	8/19 (42.11)
Urinary system involvement	7/19 (36.84)
Lactation	2/19 (10.53)
Acute pancreatitis	2/19 (10.53)
Biochemical data	
Serum total calcium	2.97 [2.38–4.50]
Serum PTH	166.4 [86.4-1914.2]
Pathology	
Number of lesions	22
Single lesion	16/19 (84.21)
Two lesions	3/19 (15.79)
Hyperplasia	1/22 (4.55)
Adenoma	18/22 (81.82)
Atypical adenoma	1/22 (4.55)
Adenocarcinoma	2/22 (9.09)
Pregnancy outcomes	
Surgery after abortion or induction of labor	5/19 (26.32)
Surgery in the third pregnancy period	1/19 (5.26)
Surgery in the middle pregnancy period	9/19 (47.37)
Fetus died in utero	1/9 (11.11)
No obvious complications	8/9 (88.89)
Surgery after cesarean section	4/19 (21.05)
US characteristics	
Number of lesions	21
Size	1.8 [0.6–7.5]
Echoes	
Hypoechoic	18/21 (85.71)
Hypoechoic with anechoic areas	3/21 (14.29)
Boundary	
Clear	20/21 (95.24)
Uncircumcised	1/21 (4.76)
Blood flow signals	
Abundant No signals	19/21 (90.48) 2/21 (9.52)

Note. — Numbers in parentheses are percentages. Numbers in the the square brackets represent ranges. MEN1, multiple endocrine neoplasia type 1; PTH, parathyroid hormone

**Table 2:** Characteristics of pregnant patients with PHPT

Patient Number	Age	Clinical manifestations	Gestation week of PHPT diagnosis	Surgery time	Operation	Pathology	Pregnancy outcome	US characteristics				
								Location	Echo	Shape	Boundary	Maximum diameter (cm)
1	26	Hyperemesis gravidarum (consciousness disorder, acute pancreatitis and HELLP syndrome)	12th week	Surgery after abortion in the 12th week	Parathyroid exploration and Parathyroidectomy	Adenocarcinoma	Abortion	Posterior of the right lobe	Hypoechoic	Regular	Clear	3.4
						Adenoma		Below the inferior pole of left lobe	Hypoechoic	Regular	Clear	1.2
2	44	Nausea and vomiting	14th week	Surgery in the 2nd trimester	Parathyroid exploration and Parathyroidectomy	Adenoma	Continuing pregnancy after surgery (Newborn without complication)	Below the inferior pole of right lobe	Hypoechoic	Regular	Clear	2.0
3	35	Bone pain; Urolithiasis	27th week	Surgery in the 2nd trimester	Parathyroidectomy	Adenoma	Continuing pregnancy after surgery (Newborn without complication)	Dorsal side of lower right lobe	Hypoechoic with anechoic	Regular	Clear	1.9
4	30	Hyperemesis gravidarum; Osteoporosis	12th week	Surgery after induction of labor in the 15th week	Parathyroid exploration and Parathyroidectomy	Adenoma	Induced labor	Below the inferior pole of right lobe	Hypoechoic	Regular	Clear	1.8
5	30	Lactation; Osteoporosis	7th week	Surgery after abortion in the 7th week	Parathyroid exploration and Parathyroidectomy	MEN1 Adenoma (behind middle right lobe)	Abortion	Behind middle right lobe	Hypoechoic	Regular	Clear	2.1
						MEN1 Adenoma (below the inferior pole of right lobe)		Not seen (false negative)				
6	38	Lactation; Osteoporosis	7th week	Surgery after delivery	Parathyroidectomy	MEN1 Adenoma (up)	Surgery after cesarean section (Newborn without complication)	Behind the upper left lobe	Hypoechoic	Regular	Clear	1.2
						MEN1 Adenoma (low)		Below the inferior pole of left lobe	Hypoechoic	Regular	Clear	1.2
7	28	Nausea and vomiting	14th week	Surgery in the 2nd trimester	Parathyroidectomy	Adenoma	Continuing pregnancy after surgery (Newborn without complication)	Dorsal side of middle right lobe	Hypoechoic with anechoic	Regular	Clear	2.0
8	29	Nausea and vomiting	16th week	Surgery in the 2nd trimester	Parathyroidectomy	Adenoma	Continuing pregnancy after surgery (Newborn without complication)	Below the inferior pole of right lobe	Hypoechoic	Regular	Clear	1.3
9	35	Nausea and vomiting; Urolithiasis	22nd week	Surgery in the 2nd trimester	Parathyroidectomy	Adenoma	Continuing pregnancy after surgery (fetus died in utero)	Below the inferior pole of left lobe	Hypoechoic with anechoic	Regular	Clear	3.1
10	29	Nausea and vomiting	19th week	Surgery after induction of labor in the 19th week	Surgery in other hospital	Adenoma (confirmed through follow-up)	Induced labor	Dorsal side of lower right lobe	Hypoechoic	Regular	Clear	0.6
11	27	Hyperemesis gravidarum; Osteoporosis	9th week	Surgery after abortion in the 9th week	Parathyroid exploration and Parathyroidectomy	Atypical adenoma, not excluding adenocarcinoma	Abortion	Dorsal side of right lobe	Hypoechoic	Irregular	Uncircumcised	5.3
12	30	Nausea and vomiting; Urolithiasis	19th week	Surgery in the 2nd trimester	Parathyroidectomy	Hyperplasia	Continuing pregnancy after surgery (Newborn without complication)	Below the inferior pole of left lobe	Hypoechoic	Regular	Clear	1.3
13	33	Nausea and vomiting	19th week	Surgery in the 2nd trimester	Parathyroidectomy	Adenoma	Continuing pregnancy after surgery (Newborn without complication)	Posterior left lobe	Hypoechoic	Regular	Clear	7.5
14	32	Nausea and vomiting	16th week	Surgery in the 2nd trimester	Parathyroidectomy	Adenoma	Continuing pregnancy after surgery (Newborn without complication	Below the inferior pole of right lobe	Hypoechoic	Regular	Clear	1.0
15	33	Nausea and vomiting	7th week	Surgery after delivery	Parathyroidectomy	Adenoma	Continuing pregnancy after surgery (Newborn without complication)	Behind the upper pole of left lobe	Hypoechoic	Regular	Clear	2.0
16	28	Hypercalcemia	8th week	Surgery after delivery	Parathyroidectomy	Adenoma	Continuing pregnancy after surgery (Newborn without complication)	Middle dorsal side of right lobe	Hypoechoic	Regular	Clear	1.6
17	32	Urolithiasis	14th week	Surgery in the 2nd trimester	Parathyroidectomy	MEN1 Adenoma	Continuing pregnancy after surgery (Newborn without complication)	Behind the inferior pole of left lobe	Hypoechoic	Regular	Clear	3.6
18	29	Arthralgia	38th week	Surgery after delivery	Parathyroidectomy	Adenocarcinoma (Well differentiated)	Surgery after cesarean section (Newborn without complication)	Middle dorsal side of right lobe	Hypoechoic	Regular	Clear	3.2
19	32	Hypercalcemia	28th week	Surgery in the 3rd trimester	Parathyroidectomy	Adenoma	Twins, fetal death in utero before surgery	Below the inferior pole of right lobe	Hypoechoic	Regular	Clear	1.8

The median age of the included patients was 30 years (range 26 to 44 years). Among these 19 patients, three were clinically diagnosed as multiple endocrine neoplasia type 1 **(**MEN1). The most common clinical manifestations were gastrointestinal symptoms, including nausea, vomiting, and anorexia (63.16%, 12/19), of which three cases were hyperemesis gravidarum. The second common symptom was bone involvement, manifested mostly as osteoporosis and bone pain (42.11%, 8/19). Two patients (10.53%) manifested as lactation; that is, two of three patients were diagnosed as MEN1. Two patients (10.53%) had consciousness disorder, acute pancreatitis, and eclampsia, and one of them had HELLP syndrome (hemolysis, elevated liver enzymes, and thrombocytopenia). The median preoperative serum total calcium in the 19 patients was 2.97 mmol/L (2.38–4.50 mmol/L), and the median serum preoperative parathyroid hormone (PTH) was 166.4 pg/mL (86.4–1914.2 pg/mL). Sixteen patients (84.21%) had hypophosphatemia.

Five patients (26.32%, 5/19) underwent surgery after an abortion in early pregnancy (*N* = 3) or induction of labor in the second trimester (*N* = 2). Most of the patients (47.37%, 9/19) underwent surgery during the second trimester and continued their pregnancy after the surgery. Of these, one fetus died in utero half a month after surgery in the second trimester. A preoperative serum PTH of 634.0 pg/mL and a preoperative serum calcium of 4.50 mmol/L were the highest among the nine cases. The other newborns had no obvious complications. One patient with twins underwent parathyroid surgery in the 29th week. Unfortunately, the patient had developed acute pancreatitis in the 28th week. Before her surgery, her two fetuses died in utero in the 27th and 29th weeks, respectively. Four of the patients underwent parathyroid surgery after a cesarean section. No abnormalities were seen in the newborns.

After surgery, the serum PTH returned to normal in all 19 patients, and there was no increase during follow-up. According to the exploratory parathyroid surgery and postoperative pathological results, there were 22 lesions in the 19 patients (three patients had two lesions), including 18 adenomas (81.82%, 18/22), one hyperplasia (4.55%, 1/22), one atypical parathyroid tumor (4.55%, 1/22), and two adenocarcinomas (9.09%, 2/22). The median size of the lesions was 1.8 cm (0.6–7.5 cm). Among them, 90.91% (20/22) of the lesions had a maximum diameter ≥ 1.0 cm. No ectopic lesions were found.

### Imaging results

All 19 patients underwent preoperative parathyroid ultrasonography. A total of 21 lesions were found by US (including two foci identified in two patients each). Referring to the surgical and histopathological findings, a total of 21 lesions were found on US. The diagnostic sensitivity of the US was 95.45% per lesion and 100% per patient. The positive predictive value was 100%. There was one false negative case on US. The patient underwent parathyroid surgery after an abortion in the 7th week. Two foci were confirmed by surgery and pathology, and the clinical diagnosis was MEN1. The adenoma behind the middle of the right lobe was found by US but the other adenoma with a maximum diameter of 0.6 cm located at the lower pole of the right thyroid lobe was missed. The latter was not found by either US or ^99m^Tc-MIBI scintigraphy before surgery.

**Fig. 1:**
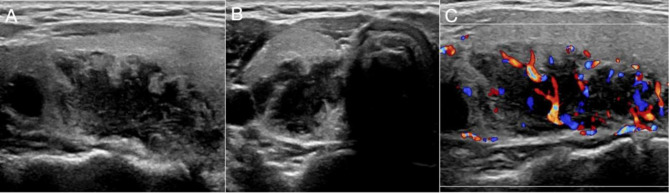
US images of a 9th week-pregnancy female, with pathological results showing an atypical parathyroid tumor. **A**.**B**. The longitudinal and cross section images showed an irregular shape and unclear boundary. **C**. Color Doppler US showed abundant blood flow signals

As for the US features, 18 lesions (85.71%) were solid, while three (14.29%) were complex cystic lesions. The mean maximum diameter of these three lesions was 2.3 cm. Twenty lesions (95.24%, 20/21) showed a regular shape and clear boundaries. One lesion showed an irregular shape with an uncircumscribed boundary and a maximum diameter of 5.3 cm. It was confirmed as an atypical parathyroid tumor by pathology and could not be excluded as adenocarcinoma (Fig. 1). Two lesions (9.52%, 2/21) showed no significant blood flow signal with maximum diameters of 1.2 cm and 0.6 cm, respectively.

Eight patients underwent ^99m^Tc-MIBI parathyroid imaging. Four of them underwent scintigraphy after an abortion or induction of labor, while the others underwent scintigraphy after delivery. All patients who chose to continue their pregnancy refused to undergo ^99m^Tc-MIBI parathyroid imaging. ^99m^Tc-MIBI examination achieved consistent results with US in six patients (6/8, 75%). However, neither US nor ^99m^Tc-MIBI scintigraphy found all foci in the MEN1 patient. In another two inconsistent cases, one patient was confirmed as having two lesions during surgery. While US found both lesions, ^99m^Tc-MIBI scintigraphy missed one lesion with a maximum diameter of 1.2 cm. One patient’s ^99m^Tc-MIBI scintigraphy result was negative, but their US showed a lesion with a maximum diameter of 2.0 cm. The sensitivity of ^99m^Tc-MIBI scintigraphy was 70% (7/10) per lesion.

## Discussion

PHPT in pregnancy can increase the risk of maternal and infant complications. It is suggested that pregnant women with PHPT should be managed by a multidisciplinary team [13, 14]. A systematic review of observational studies including 382 women with gestational PHPT, of whom 108 had undergone parathyroidectomy during pregnancy, reported a significantly lower infant complication rate for surgery versus medical therapy (9.1% vs. 38.9%), with similar results when restricting the analysis to asymptomatic cases [15]. This indicates that surgical treatment is effective and safe during pregnancy and for neonatal outcome. Therefore, preoperative localization of lesions plays a crucial role in achieving surgical success. However, in China, ^99m^Tc-MIBI scintigraphy was not accepted by PHPT patients who wish to continue their pregnancy, although European expert consensus consider it feasible [10]. 

Our study enrolled a large number of patients of PHPT during pregnancy and showed that preoperative US localization of lesions had a high sensitivity (100%, 19/19) and positive predictive value (100%) in such a specific population. Nine patients underwent surgery during the second trimester, eight of which had a successful surgery with no complications in their newborns. That is, accurate preoperative lesion localization and appropriate timing of surgery can significantly improve pregnancy outcomes. Compared with previous literature on non-pregnant PHPT populations, our study presented the characteristics of higher blood calcium and PTH levels and larger size of parathyroid lesions [16, 17]. Therefore, we identified 95.45% (21/22) of the lesions in US. More detailed comparisons can be made through case-control studies in the future.

In our cohort, US had a significantly high sensitivity for preoperative lesion localization. Lesion size could be a contributing factor to imaging localization efficacy. With an increase in lesion size, the sensitivity of the imaging examinations also increased [17]. In our cohort, the median maximum diameter of the parathyroid lesions was 1.8 cm (range 0.6–7.5 cm) and 90.91% were over 1.0 cm. This could help explain the outstanding performance of US.

For most cases, ectopic glands are another challenge for localization [18]. The proportion of ectopic adenomas in adult patients is 6–16% [19]. The sensitivity of US varies depending on the location of the parathyroid lesion. Its overall sensitivity has been reported to be 55–87% and is especially low in cases with ectopic parathyroid tissues or norm calcemic PHPT [20–22]. In our group, no ectopic location was reported according to the surgical findings, which could also explain the high sensitivity of the US examination.

Finally, multiple-gland disease is one of the most difficult situations for preoperative localization imaging. It is widely accepted that there is an association between multifocal lesions and MEN1 in the pediatric population. MEN1 patients may have a higher incidence of multifocal lesions [23, 24]. In our study, three patients (15.79%) were clinically diagnosed as MEN1 and two of them had two lesions.

Avoiding radiation exposure in pregnancy is critical for guiding decisions on the choice of imaging methods for PHPT. In our cohort, a total of 8 patients underwent ^99m^Tc-MIBI scintigraphy, including 4 after an abortion or induction of labor and 4 after delivery. Although the European expert consensus states that ^99m^Tc-MIBI scintigraphy is an acceptable option [10], our patients who choose to continue pregnancy refuse to undergo ^99m^Tc-MIBI examination. In our cohort, a high positive rate (100%) of preoperative localizing parathyroid lesion(s) was achieved by US. Thus, it is not recommended to use ^99m^Tc-MIBI scintigraphy as a routine procedure for localizing PHPT lesions during pregnancy.

Our study had several limitations. First, due to the low incidence of PHPT in pregnancy, the number of enrolled cases in this study was relatively small. However, it currently includes the largest number of reported cases of an imaging study. Second, this was a retrospective study conducted at a single academic institution. Therefore, selection bias might have occurred. and it was difficult for the radiologists to be completely blinded to imaging examinations before reaching a diagnosis. In addition, owing to the limited acceptance of ^99m^Tc-MIBI scintigraphy examination among pregnant women, US emerged as the sole preoperative localization technique in our cohort. For those patients yielding negative US findings and decline to undergo the ^99m^Tc-MIBI scintigraphy examination, no surgical exploration was conducted. Consequently, we are unable to procure the pathological results serving as the gold standard, nor can we include this subset of patients in our study. This circumstance may potentially lead to an overestimation of the diagnostic efficacy of US in pregnancy-associated PHPT.

## Conclusions

Parathyroid US is an accurate preoperative localization imaging modality in pregnant women with PHPT and is indicated for surgery. Curative surgery performed in the second trimester improved the patients’ pregnancy outcomes.

## Electronic supplementary material

Below is the link to the electronic supplementary material.

Supplementary Material 1

## Data Availability

The data generated during the current study are available from the corresponding author on reasonable request.
