# Supplementary material for: Primary hyperparathyroidism during pregnancy: ultrasound as an accurate preoperative localization imaging modality
Source: Orphanet J Rare Dis. 2025 Mar 31;20:148. doi: 10.1186/s13023-024-03519-w (PMC11960009; doi:10.1186/s13023-024-03519-w)
Supplement: Supplementary file 1 — Supplementary Material 1 [file 13023_2024_3519_MOESM1_ESM.docx]

Dear Editor,

Thank you for allowing us to submit a revised version of the manuscript “Primary Hyperparathyroidism During Pregnancy: Ultrasound as an Accurate Preoperative Localization Imaging Modality” (OJRD-D-24-00238) for publication in the *Orphanet Journal of Rare Diseases*. We appreciate you and the reviewers dedicating your valuable time to reviewing our paper and providing insightful comments. Your valuable comments led to possible improvements to the current version. We have carefully considered the comments and tried our best to address every comment. Below we provide the point-by-point responses. Reviewers’ comments are in bold type, followed by our responses and edits. All modifications in the manuscript have been highlighted in yellow.

Reviewer #1: 
***The explanation of the research in the introduction is quite weak and does not adequately explain the characteristics and reasons for acquiring them. More brief and simple contents are in the introduction and discussion sections. There are numerous spelling errors in the manuscript and reference section. These must be corrected.***

Response: Thank you for your thoughtful suggestion. As per your suggestion, we have added relevant content and rewrite the “Introduction” as follows:

Primary hyperparathyroidism (PHPT) in pregnancy is rare, accounting for roughly 1% of the total PHPT patients. However, PHPT in pregnancy can increase the risk of maternal and infant complications, including hyperemesis gravidarum, pre-eclampsia, neonatal hypocalcemia convulsion, even life-threatening complications such as abortion or stillbirth, and neonatal death. ^(1-4)^ It is important to early recognize the entity because parathyroidectomy during mid-pregnancy has been proven to improve the prognosis of pregnant women with PHPT.^(5-7)^ The precise preoperative localization of the abnormal parathyroid lesions is crucial for the success of the surgery.

Widely accepted first-line preoperative localization imaging examinations include neck ultrasound (US) and technetium-99m sestamibi (^99m^Tc-MIBI) scintigraphy. ^(8, 9)^ However, the choice of imaging protocol was essentially based on the following elements in a pregnant woman: reduction of radiation of the fetal thyroid and whole body as low as reasonably possible, and ensuring the most sensitive imaging possible.^(10, 11)^ US has been widely used for preoperative evaluation because of the advantages of non-radiation and ease of use and convenience. However, there is still a lack of evidence on whether US can be accurately used as the single imaging modality for preoperative localization with the intent to minimize nuclide exposure in pregnant patients.

Our study aimed to evaluate the diagnostic performance of parathyroid US as the primary imaging modality in evaluating PHPT in pregnant women. Our secondary aim was to evaluate the added benefit of parathyroid radionuclide imaging.

We are very sorry for the minor spelling errors in our manuscript, and we have carefully checked and made corrections throughout the revised version.

Reviewer #2:  ***Congratulations on this well-written study reporting the value of ultrasound in a rare group of patients who had pregnancy and primary hyperparathyroidism. They showed a very high diagnostic sensitivity of US as compared to surgery and histopathology, confirming its value of localization of parathyroid lesions in these patients.***

***Some specific comments:
M&M
1.    the diagnostic criteria of PHPT should be more detailed, such as the level of PTH, how to exclude secondary hyperparathyroidism, etc.***

Response: Thank you for your kind reminder. Following your suggestion, we have added the detailed diagnostic criteria of PHPT into Methods as follows：

PHPT was defined as hypercalcemia with parathyroid hormone (PTH) levels higher than normal or not suppressed; or normocalcemic PHPT as a persistently normal total albumin corrected and ionized serum calcium concentrations and persistently elevated PTH levels after ruling out secondary causes of hyperparathyroidism such as renal disease, hypovitaminosis D, and hypercalciuria. ^(12)^

The diagnosis of PHPT with pregnancy was determined as PHPT diagnosed during pregnancy or pregnancy occurring in PHPT patients with uncontrolled conditions. ^(10)^

***2.    please define "incomplete imaging" in the exclusion criteria.***

Response: Thank you for your thoughtful reminder. Following your suggestion, we have modified and added relevant contents in the section of “Methods” as follows:

The inclusion criteria were PHPT patients with pregnancy who had undergone surgery and had the definite pathological results. If patients did not have preoperative US examination, and/or 6-month postoperative laboratory values, they were excluded from analysis.

***3.    please define the time of follow-up and the information to collect.***

Response: Thank you for your kind reminder. As per your suggestion, we have added the following contents in “Methods”:

In addition, the postoperative laboratory values were collected including PTH and blood calcium at least 6 months after parathyroidectomy. The pregnancy outcomes were also obtained.

***4.    Imaging protocol, you stated that "Per the policy at our institution for PHPT, parathyroid US and 99mTc-MIBI SPECT/CT should be performed for patients with suspected clinical symptoms. Does it mean that patients have to do both examinations? or one of the two?***

Response: Thank you for your thoughtful suggestion. We have modified the following contents in “Methods”:

Per the policy at our institution for PHPT, parathyroid US was recommended as the first-line imaging examination. After a thorough communication, the patient would decide whether to undergo ^99m^Tc-MIBI scintigraphy.

***Results
1.    The gestation week of PHPT diagnosis should be provided.***

Response: Thank you for your kind reminder. Following your suggestion, we have added the relevent contents in Table 2.

**Table 2** Characteristics of pregnant patients with PHPT

| Patient Number | Age | Clinical manifestations | Gestation week of PHPT diagnosis | Surgery time | Operation | Pathology | Pregnancy outcome | US characteristics | | | | |
| --- | --- | --- | --- | --- | --- | --- | --- | --- | --- | --- | --- | --- |
|  |  |  |  |  |  |  |  | Location | Echo | Shape | Boundary | Maximum diameter (cm) |
| 1 | 26 | Hyperemesis gravidarum (consciousness disorder, acute pancreatitis and HELLP syndrome) | 12^th^ week | Surgery after abortion in the 12th week | Parathyroid exploration and Parathyroidectomy | Adenocarcinoma | Abortion | Posterior of the right lobe | Hypoechoic | Regular | Clear | 3.4 |
|  |  |  |  |  |  | Adenoma |  | Below the inferior pole of left lobe | Hypoechoic | Regular | Clear | 1.2 |
| 2 | 44 | Nausea and vomiting | 14^th^ week | Surgery in the 2^nd^ trimester | Parathyroid exploration and Parathyroidectomy | Adenoma | Continuing pregnancy after surgery (Newborn without complication) | Below the inferior pole of right lobe | Hypoechoic | Regular | Clear | 2.0 |
| 3 | 35 | Bone pain; Urolithiasis | 27^th^ week | Surgery in the 2^nd^ trimester | Parathyroidectomy | Adenoma | Continuing pregnancy after surgery (Newborn without complication) | Dorsal side of lower right lobe | Hypoechoic with anechoic | Regular | Clear | 1.9 |
| 4 | 30 | Hyperemesis gravidarum; Osteoporosis | 12^th^ week | Surgery after induction of labor in the 15th week | Parathyroid exploration and Parathyroidectomy | Adenoma | Induced labor | Below the inferior pole of right lobe | Hypoechoic | Regular | Clear | 1.8 |
| 5 | 30 | Lactation; Osteoporosis | 7^th^ week | Surgery after abortion in the 7th week | Parathyroid exploration and Parathyroidectomy | MEN1  Adenoma (behind middle right lobe) | Abortion | Behind middle right lobe | Hypoechoic | Regular | Clear | 2.1 |
|  |  |  |  |  |  | MEN1  Adenoma (below the inferior pole of right lobe) |  | Not seen (false negative) | | | | |
| 6 | 38 | Lactation; Osteoporosis | 7^th^ week | Surgery after delivery | Parathyroidectomy | MEN1  Adenoma (up) | Surgery after cesarean section (Newborn without complication) | Behind the upper left lobe | Hypoechoic | Regular | Clear | 1.2 |
|  |  |  |  |  |  | MEN1  Adenoma (low) |  | Below the inferior pole of left lobe | Hypoechoic | Regular | Clear | 1.2 |
| 7 | 28 | Nausea and vomiting | 14^th^ week | Surgery in the 2^nd^ trimester | Parathyroidectomy | Adenoma | Continuing pregnancy after surgery (Newborn without complication) | Dorsal side of middle right lobe | Hypoechoic with anechoic | Regular | Clear | 2.0 |
| 8 | 29 | Nausea and vomiting | 16^th^ week | Surgery in the 2^nd^ trimester | Parathyroidectomy | Adenoma | Continuing pregnancy after surgery (Newborn without complication) | Below the inferior pole of right lobe | Hypoechoic | Regular | Clear | 1.3 |
| 9 | 35 | Nausea and vomiting; Urolithiasis | 22^nd^ week | Surgery in the 2^nd^ trimester | Parathyroidectomy | Adenoma | Continuing pregnancy after surgery (fetus died in utero) | Below the inferior pole of left lobe | Hypoechoic with anechoic | Regular | Clear | 3.1 |
| 10 | 29 | Nausea and vomiting | 19^th^ week | Surgery after induction of labor in the 19th week | Surgery in other hospital | Adenoma (confirmed through follow-up) | Induced labor | Dorsal side of lower right lobe | Hypoechoic | Regular | Clear | 0.6 |
| 11 | 27 | Hyperemesis gravidarum; Osteoporosis | 9^th^ week | Surgery after abortion in the 9th week | Parathyroid exploration and Parathyroidectomy | Atypical adenoma, not excluding adenocarcinoma | Abortion | Dorsal side of right lobe | Hypoechoic | Irregular | Uncircumcised | 5.3 |
| 12 | 30 | Nausea and vomiting; Urolithiasis | 19^th^ week | Surgery in the 2^nd^ trimester | Parathyroidectomy | Hyperplasia | Continuing pregnancy after surgery (Newborn without complication) | Below the inferior pole of left lobe | Hypoechoic | Regular | Clear | 1.3 |
| 13 | 33 | Nausea and vomiting | 19^th^ week | Surgery in the 2^nd^ trimester | Parathyroidectomy | Adenoma | Continuing pregnancy after surgery (Newborn without complication) | Posterior left lobe | Hypoechoic | Regular | Clear | 7.5 |
| 14 | 32 | Nausea and vomiting | 16^th^ week | Surgery in the 2^nd^ trimester | Parathyroidectomy | Adenoma | Continuing pregnancy after surgery (Newborn without complication | Below the inferior pole of right lobe | Hypoechoic | Regular | Clear | 1.0 |
| 15 | 33 | Nausea and vomiting | 7^th^ week | Surgery after delivery | Parathyroidectomy | Adenoma | Continuing pregnancy after surgery (Newborn without complication) | Behind the upper pole of left lobe | Hypoechoic | Regular | Clear | 2.0 |
| 16 | 28 | Hypercalcemia | 8^th^ week | Surgery after delivery | Parathyroidectomy | Adenoma | Continuing pregnancy after surgery (Newborn without complication) | Middle dorsal side of right lobe | Hypoechoic | Regular | Clear | 1.6 |
| 17 | 32 | Urolithiasis | 14^th^ week | Surgery in the 2^nd^ trimester | Parathyroidectomy | MEN1  Adenoma | Continuing pregnancy after surgery (Newborn without complication) | Behind the inferior pole of left lobe | Hypoechoic | Regular | Clear | 3.6 |
| 18 | 29 | Arthralgia | 38^th^ week | Surgery after delivery | Parathyroidectomy | Adenocarcinoma (Well differentiated) | Surgery after cesarean section (Newborn without complication) | Middle dorsal side of right lobe | Hypoechoic | Regular | Clear | 3.2 |
| 19 | 32 | Hypercalcemia | 28^th^ week | Surgery in the 3^rd^ trimester | Parathyroidectomy | Adenoma | Twins, fetal death in utero before surgery | Below the inferior pole of right lobe | Hypoechoic | Regular | Clear | 1.8 |

***Discussion
1.    Should discuss a bit the difference of US characteristics of parathyroid lesions between pregnant women and other non-pregnant patients. Also, the sensitivity and specificity of US in identifying parathyroid lesions should be compared to previous reports.***

Response: Thank you for your kind reminder. As per your suggestion, we have added the following contents in Discussion:

Compared with previous literature on non-pregnant PHPT populations, our study presented the characteristics of higher blood calcium and PTH levels and larger size of parathyroid lesions.^(16,17)^ Therefore, we identified 95.45% (21/22) of the lesions in US. More detailed comparisons can be made through case-control studies in the future.

***2.    Is it possible that the high sensitivity of US is because that US is the only imaging tool? And US negative patients were not included? Please elaborate on the selection bias of this study.***
Response: Thank you for your thoughtful suggestion. We agree with your viewpoint and we have added the following contents in “Limitation”:

In addition, owing to the limited acceptance of ^99m^Tc-MIBI scintigraphy examination among pregnant women, US emerged as the sole preoperative localization technique in our cohort. For those patients yielding negative US findings and decline to undergo the ^99m^Tc-MIBI scintigraphy examination, no surgical exploration was conducted. Consequently, we are unable to procure the pathological results serving as the gold standard, nor can we include this subset of patients in our study. This circumstance may potentially lead to an overestimation of the diagnostic efficacy of US in pregnancy-associated PHPT.

We appreciate your valuable efforts in improving our work. We sincerely hope the revisions in the manuscript and our accompanying responses will make our manuscript suitable for *Orphanet Journal of Rare Diseases*. We welcome any further comments or suggestions you may have. We look forward to hearing from you.

Yours sincerely,

Qingli Zhu

E-mail: [zqlpumch@126.com](mailto:zqlpumch@126.com)

Ou Wang

E-mail: wang_ou2010@126.com
